# Response to genes that improved fitness also cost modern humans: evidence for genes with antagonistic effects on longevity and disease

**DOI:** 10.1093/emph/eoz003

**Published:** 2019-01-23

**Authors:** Steven N Austad, Jessica M Hoffman

**Affiliations:** Department of Biology, University of Alabama at Birmingham, Birmingham, AL, USA

Byars and Voskarides, responding to our review of empirical support for George Williams’ antagonistic pleiotropy (AP) theory of the evolution of aging [1], feel that we have ‘failed to acknowledge’ recent human studies supporting the theory. Indeed, we mentioned no human studies because we had intended our review to present only the strongest evidence supporting the theory which has been done almost entirely in laboratory model organisms. For this reason, while we mentioned a few studies from natural populations, we emphasized how such nonexperimental studies could be *consistent* with the AP mechanisms, but could not be *cleanly attributed* to it. Thus, we focused on experimental studies—those in which *experimental* manipulation of a single gene had clear antagonistic effects on fitness components in early versus late life as Williams predicted. Experimental studies establish cause-and-effect in a way that correlational studies such as those cited by Byars and Voskarides cannot.

It is an unfortunate truth about research on humans that because experimental studies are often impossible, results are almost inevitably correlational, which in our view makes virtually any single study highly suggestive at best, but never compelling. To illustrate why, we consider one of the studies adduced by Byars and Voskarides, although we could have chosen any of the others. That study identifies numerous human alleles (or Single nucleotide Polymorphisms) pre-disposing individuals to coronary artery disease (CAD) but also conferring reproductive advantages early in life [2]. As to the nature of the evidence they presented, they identified a correlation, e.g. signs of positive selection in genomic regions associated with CAD across a number of modern populations, followed by an assumption, that CAD significantly manifested during reproductive years over the course of the evolutionary history of humans, plus another correlation and a further assumption, viz. genomic associations of CAD-related regions with lifetime reproductive success (LRS) in a modern, middle class, overwhelmingly white population represents the same or similar genomic associations with LRS across human evolutionary history.

This is a very nice study given the limitations of human research. The best that could be done with available data was done. Note, however, that one of the first lessons of statistical reasoning is that correlation does not equal causation and, yes, genomic associations are correlations. Further, their assumption about the early life CAD across human evolutionary history is supported by evidence from modern populations plus a study of the existence and extent of vascular calcification as revealed by whole body CT scans in modern Egyptians and Egyptian mummies who lived as long as 5000 years ago [3]. Again, this is plausible. However, counterevidence is available from the Tsimane of eastern Bolivia, people living a pre-industrial lifestyle with a subsistence diet and chronically high levels of physical activity. The Tsimane when examined using the same whole body CT scans by the same group as scanned the Egyptians mentioned above found that only 1 and 2% of Tsimane aged 45–54 and 55–64 years, respectively, had sufficient arterial calcification to be considered at moderate or high risk of CAD [4]. So the assumption about CAD’s impact among early humans has mixed evidentiary support.

Our point in noting these things is certainly not to denigrate Byars *et al.*’s study or the other studies cited by the authors. These are some very fine studies using state-of-the-art genomic analyses. We simply wanted to explain why we consider such studies less compelling as support for Williams’ AP theory than experimental studies done in model laboratory organisms with specific and purposeful manipulation of specific individual genes.

**Conflict of interest:** None declared.
